# P-823. Blood Culture Stewardship: An Essential Practice for CLABSI Reduction

**DOI:** 10.1093/ofid/ofaf695.1031

**Published:** 2026-01-11

**Authors:** Ronald A Besandre, April McDougal, Rachel S Britt, Ryan S Ferren, Noor Zaidan, David Reynoso

**Affiliations:** University of Texas Medical Branch, League City, Texas; University of Texas Medical Branch, League City, Texas; UTMB Health, Galveston, Texas; UTMB Health, Galveston, Texas; UTMB, Galveston, Texas; The University of Texas Medical Branch, Galveston, Texas

## Abstract

**Background:**

Blood cultures facilitate management of infections like endocarditis and catheter-line associated bloodstream infections (CLABSIs). However, false positives misdiagnosed as true bacteremia enable unnecessary antibiotics and costly hospitalizations. To decrease blood culture contamination (BCC) and CLABSIs, we developed a blood culture collection kit, effecting moderate improvement in the year following kit roll-out. Nationwide shortage of blood culture media bottles in 2024 provided further opportunity to improve the quantity and quality of blood culture orders and decrease CLABSI rates.

**Table 1.**
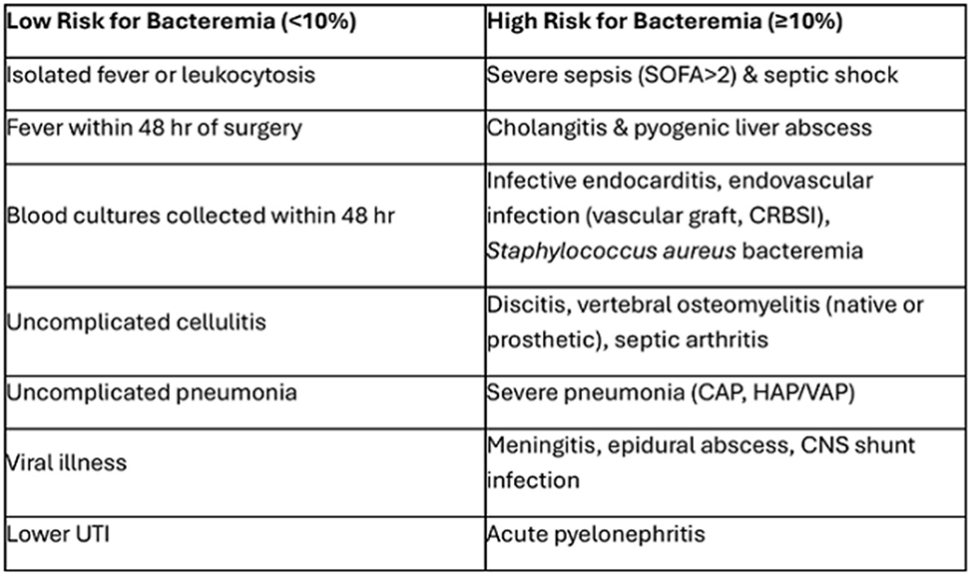
Pretest probability of bacteremia (Adapted from CID. 2020 Aug22;71(5):1339-1347)

**Figure 1.**
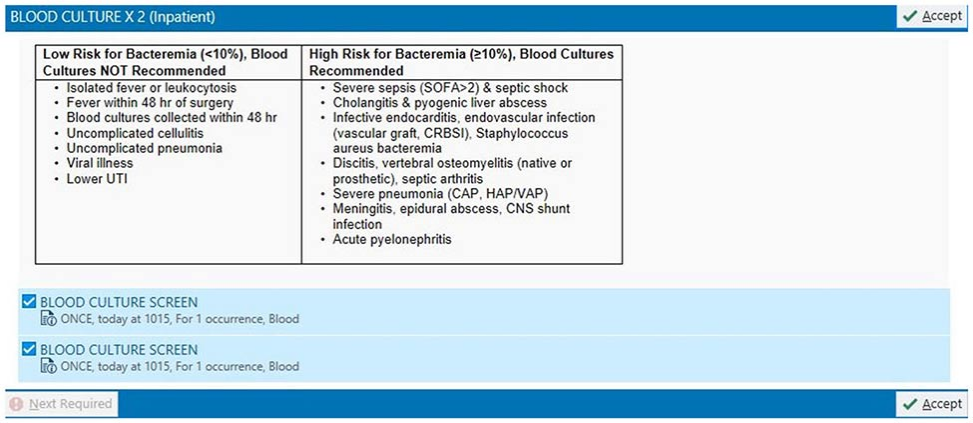
EMR guidance prompting user to consider bacteremia risk by condition.

**Methods:**

Blood culture stewardship education about pre-test probability of bacteremia was provided via broadcast emails and focused lectures to faculty and house staff in September 2024 (Table 1). In November 2024, guidance indicating conditions with high pre-test probability of bacteremia was added to computerized provider order entry (CPOE) for blood cultures (Figure 1).

**Results:**

Before intervention, an average of 2896 blood cultures were ordered monthly across our health system (145 orders per 1000 patient days), decreasing to 1909 monthly blood culture orders post-intervention, or 95 orders per 1000 patient days, for an overall 35% year-to-year reduction. True bacteremia rates before and after intervention were 20% and 23%, respectively. In CY23 Q4 (blood culture collection kit alone), 8 CLABSIs occurred, compared with 2 CLABSIs after interventions (75% reduction) in CY24 Q4. Overall, 43 vs 29 CLABSIs occurred in CY23 vs CY24, deriving a 32.5% reduction. Throughout this time, systemwide BCC averaged 2.4% for CY24, sustaining our < 3% goal.

**Conclusion:**

The quality and quantity of blood culture orders improved through collaborative multidisciplinary efforts including focused education and CPOE optimization, without a decrease in true bacteremia detection. These efforts have also positively affected CLABSI events.

**Disclosures:**

All Authors: No reported disclosures

